# Hepatic metastasis of thymoma: case report and immunohistochemical study

**DOI:** 10.3332/ecancer.2016.693

**Published:** 2016-11-22

**Authors:** Daniela Speisky, María Teresa García de Davila, Felix Vigovich, Julian Mendez, Rafael Maurette, Marcos García Ejarque, Juan Carlos Spina, Alejandro Iotti, Pablo Dezanzo

**Affiliations:** 1 Department of Histopathology, Hospital Británico, Buenos Aires C1280AEB, Argentina; 2 Department of Hepatobiliary Surgery, Hospital Británico, Buenos Aires C1280AEB, Argentina; 3 Department of Diagnostic Imaging, Hospital Británico, Buenos Aires C1280AEB, Argentina

**Keywords:** thymoma, metastasis of thymoma, hepatic metastasis

## Abstract

Thymomas are rare tumours characterised by their slow growth and capacity to invade directly by contiguity. While distant dissemination is infrequent, all sub-types of thymoma have the capacity to metastasise to extrathoracic organs.

We present here the case of a female patient with a liver mass discovered 13 years after the removal of a mediastinal thymoma and after ten years from thyroidectomy for papillary carcinoma.

The histopathological study showed that the lesion contained an epithelial component, which was immunohistochemically positive for pankeratin. It was accompanied by numerous small lymphocytes testing positive for TdT, CD3, CD4, CD5, CD8, CD99, and CD43. The result was consistent with hepatic metastatic thymoma sub-type B1, according to the World Health Organisation classification (WHO).

Our case highlights the importance of morphological and immunohistological examinations in the differential diagnosis of visceral masses in patients with a history of thymoma. Given the infrequency of its metastasis and the increased risk of developing other primary tumours that these patients have, these studies play a significant role.

## Introduction

Thymomas are epithelial tumours with thymic differentiation that are accompanied by non-neoplastic lymphocytes [1]. While their frequency compared to other tumours is very low, they should be noted in the differential diagnosis of anterior mediastinal masses since they make up approximately 40% of tumours in this area [2].

These neoplastic masses are characterised by their slow growth and capacity to invade adjacent organs by contiguity [2]. By contrast, the occurrence of distant metastasis is less frequent, and in the majority of cases it is related to thymic carcinomas [3, 4].

Numerous classifications have tried to correlate the different histological sub-types of thymic tumours with their clinical behaviour and prognosis [5, 6]. Nevertheless, these systems of classification reflect the confusion that exists around these neoplasms, making it difficult to compare the different reported series [7]. Recently, epithelial thymic tumours were re-classified by the World Health Organisation (WHO) as type A, AB, B1, B2, B3 thymomas and thymic carcinomas according to the predominance of the epithelial or lymphocytic component [1, 2, 8]. In that classification, the different sub-types of thymic tumours are arranged in increasing order of malignancy, although it has been demonstrated that all of them, and not only the carcinomas, have the capacity to metastatise.

## Clinical case

**Figure 1.**
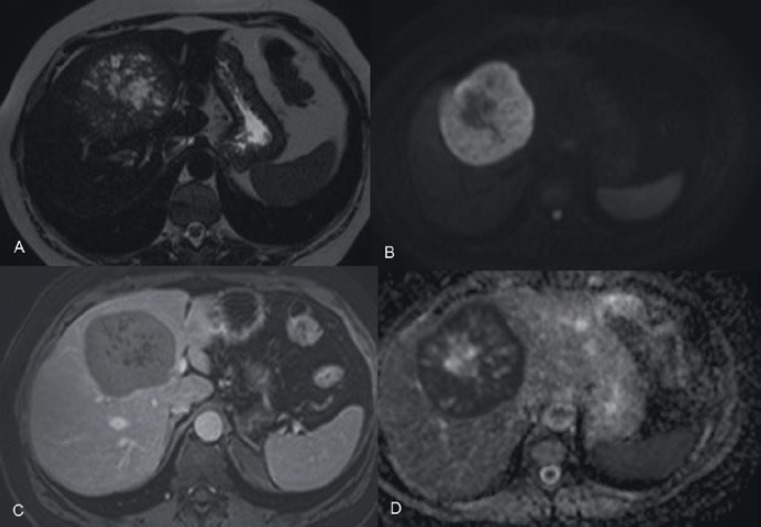
MRI of the abdomen with EV contrast. An expansive formation in segment VIII and spreading towards segment IVA. It is heterogeneously T2-hyperintense (A) and in diffusion sequences (B), with marked hypointensity in the apparent diffusion coefficient (ADC) maps (D), as manifestation of increased cellularity. An endovenous contrast injection faintly highlighted a peripheral capsule in the later cuts (C). Ref: MRI: magnetic resonance imaging. EV: endovenous.

A 55 year old woman presented with nonspecific progressive discomfort in the right hypochondrium dating back two months. Additional studies were carried out. An ultrasound scan showed a hepatic nodule of 92 mm in widest diameter in segments IV, V, and VIII, while the results of all the other exams were normal. This was supplemented by a computerised axial tomography (CAT) scan which revealed a heterogeneous formation of 77 mm x 76 mm with hypodense areas in its interior, and a peripheral enhancement, located in hepatic segment VIII. The magnetic resonance imaging (MRI) revealed an expansive well-defined heterogeneous formation 81 x 72 mm, T1-hypointense, and T2-hyperintense with restricted diffusion. An endovenous contrast injection faintly highlighted a peripheral capsule in later cuts (Figure 1).

We reviewed the patient’s medical history, which revealed that 13 years earlier she had undergone surgical intervention for a 10 cm mediastinal tumour, which was completely resected. The histopathological findings were consistent with a thymoma surrounded by an undamaged fibrous capsule. Three years later, the patient underwent a total thyroidectomy on account of a thyroid papillary carcinoma. There was no other relevant medical history data.

Given the clinical and imagenological findings described above, it was decided to surgically remove hepatic segments IV, V, and VIII.

**Figure 2.**
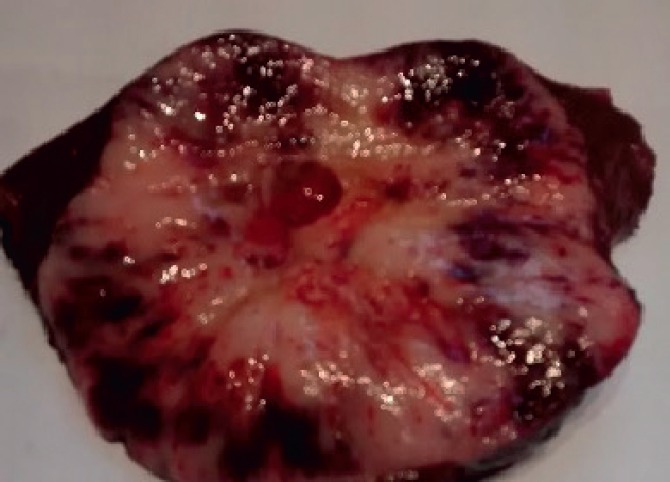
Macroscopic examination of hepatic segments IV, V, and VIII. Presence of a solid, whitish tumour with central cystic degeneration measuring 9 × 9 cm.

**Figure 3.**
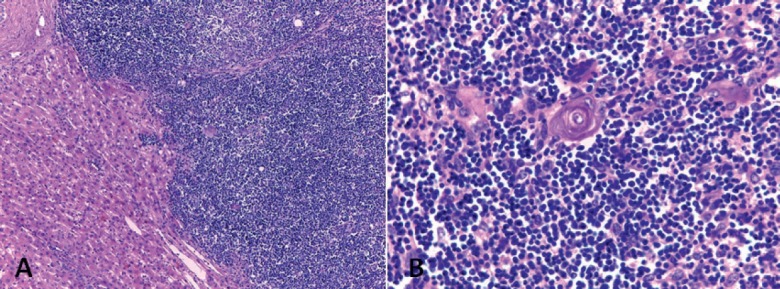
Hepatic metastatic thymoma sub-type B1, stained with haematoxylin and eosin. A) The lesion is organotypic, with a scarcity of epithelial cells and an abundance of small lymphocytes with an expansive growth pattern. B) Epithelial and lymphocytic component of the thymoma. Original magnification 100 × (A); 400 × (B).

The macroscopic examination showed a solid, whitish tumour with hemorrhagic and cystic degeneration of 9 x 9 cm. This was found to be surrounded by a thin capsule (Figure 2). The histological sections of material, embedded in paraffin and coloured with haematoxylin and eosin, showed that the lesion comprised numerous small, monomorphic lymphocytes, without cytological atypia. Among these were also seen occasional cells with large eosinophilic cytoplasm and nuclei with finely granular chromatin. In addition, we saw structures that mimicked Hassall’s corpuscles and perivascular spaces in a stockade of epithelioid cells around the vessels. The lesion presented an expansive border which delineated it from the adjacent hepatic parenchyma, which itself showed no significant changes (Figures 3A and 3B).

The immunohistological techniques were carried out on histological sections of 3 microns by means of an automated system in accordance with the manufacturer’s guidelines (Benchmark XT, Ventana). Table 1 shows the immunoprofile of the epithelial and lymphocyte components of the case and the monoclonal antibody used.

**Figure 4.**
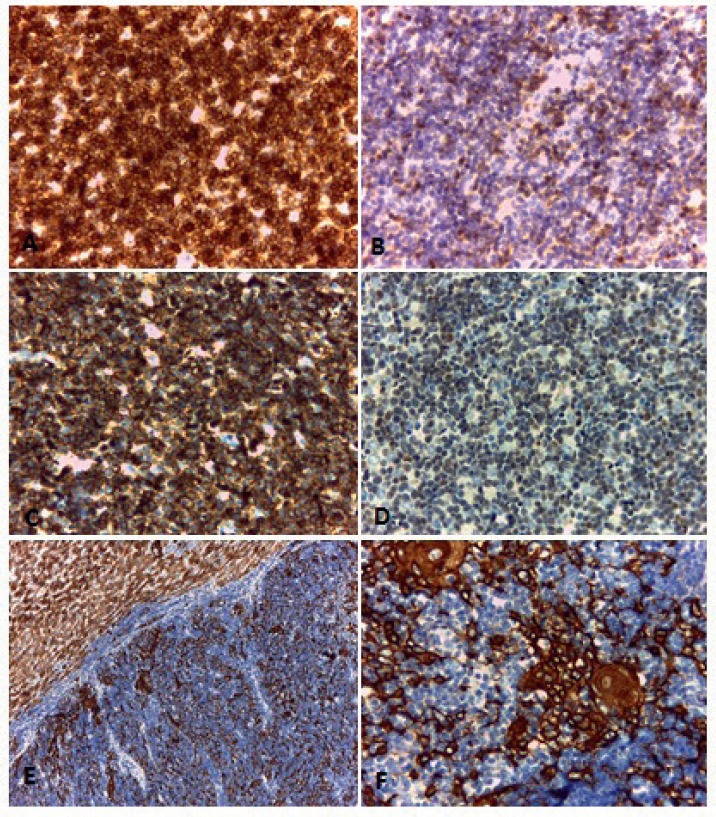
Immunohistochemical techniques. Positivity with CD3 (A), CD5 (B), CD99 (C) and TdT (D) in the lymphocytic component of the thymoma. (E and F) Positivity with pankeratin (AE1AE3) in the epithelial component of the tumour.

The lymphoid population showed intense positivity for CD3, CD5, CD8, and CD99 (Figures 4A to 4D) and weak positivity for CD4 and CD43. The cytokeratin cocktail showed positivity in the tumour’s epithelial component (Figures 4E and 4F).

Staining for the Epstein-Barr virus as well as CD30, CD15 and B lineage markers including follicular differentiation (PAX5, CD20, CD10 and BCL6) were found to be negative. There was no overexpression of protein p53.

The morphological picture in junction with the immunoprofile and the patient’s medical history allowed the diagnosis of hepatic metastatic thymoma of sub-type B1, according to the WHO classification.

At present, the patient is being regularly monitored and is free of illness six months after hepatic excision.

## Discussion

Thymomas and thymic carcinomas are rare epithelial tumours that represent fewer than 1% of all neoplasms [1, 9]. Much less frequent are distant metastases of these tumours, diagnosis of which is difficult due to their low index of suspicion and to their morphology which can appear similar to other tumours [2].

We present the case of a patient with hepatic metastasis of a sub-type B1 thymoma, according to the WHO classification. Given the infrequency of thymoma metastasis in the liver together with the long period that had passed since the excision of the primary mediastinal tumour (13 years), a thymoma metastasis was not suspected.

In this case, the immunohistological findings were relevant. The presence of small lymphocytes, without cytological atypia accompanied by isolated cells with an epithelioid appearance that mimicked Hassall’s corpuscles were the key to the diagnosis.

The immunohistochemical techniques were of considerable use, allowing us to dismiss other diagnosis such as T-lymphoblastic lymphoma in which the immunoprofile of the lymphocytes is similar, but they lack the epithelial component [3, 10].

Furthermore, the tumour growth, at both the macroscopic as well as the histological level, helped us to dismiss hepatic T-cell lymphoproliferative disorders in which the infiltration is usually diffuse and/or intrasinusoidal.

It should be stressed that in certain thymomas with a predominantly lymphocytic component, the epithelial cells can be difficult to identify and can even resemble reactive histiocytes frequently found in lymphomas [10]. In these cases, cytokeratin staining is a very valuable tool.

One should also bear in mind in the differential diagnosis, the possibility of discovering primary tumours of other lineages or even metastatic ones. The literature has demonstrated that patients with thymomas show a higher risk of developing secondary extrathymic neoplasia with a frequency between 8 and 28% [12, 13].

In this case, the patient presented with an additional history of thyroid papillary carcinoma, of which the morphology and immunoprofile are very different, so ruling out metastasis of that tumour was relatively simple. Nevertheless, due to the diversity of neoplasms associated with thymomas, certain cases of greater complexity could lead to diagnostic problems (12–13]. Different studies vary widely in the location of secondary neoplasms associated with thymic tumours. Engels *et al* found that non-Hodgkin’s lymphomas, soft-tissue sarcomas, and digestive tumours are more frequent, while Welsh *et al* described colorectal, pulmonary, breast and thyroid tumours are more common [14, 15].

Studying a broad range of thymomas, Filosso *et al* observed an increased risk of secondary synchronous and metachronous neoplasms even prior to the diagnosis of thymoma. In that study, 16.5% of patients studied for thymomas or thymic carcinomas presented with a secondary neoplasm, this being of thyroid origin in 1.9% of cases and the majority of them being metachronous, similar to the case presented here [12].

The diversity of reported findings across the different studies could be related to the rarity of these lesions with the consequent limitation in the analysed series [7]. New studies are needed to clarify the behaviour of thymic neoplasms.

## Conclusion

Even though thymomas are considered to be slow-growing tumours with predominantly local recurrences, distant metastasis of any sub-type of thymoma needs to be suspected in patients with medical history of those tumours [2, 7]. We have presented a case of hepatic metastatic thymoma occurring 13 years after the excision of a primary mediastinal tumour and ten years after a thyroidectomy for papillary carcinoma. We emphasise the importance of a morphological and immunohistological examination for the correct diagnosis of these lesions owing to their rarity and the increased risk that these patients have of developing second primary tumours [12]. In these cases, clinical monitoring, communication with the patient, and a familial study would be of great importance.

## Figures and Tables

Original magnification: 100 × (E); 400 × (A, B, C, D, F).

**Table 1.** Immunohistochemical expression of the epithelial and lymphocytic components of the thymoma. Ref: (-) negative staining; (+) weak positive staining; (++) intense positive staining. EBV: Epstein Barr virus; CQ: keratin cocktail.

Antibodies	Epithelial cells	Lymphocytes
Cytokeratin AE1AE3 (Cell Marque; CQ; mouse monoclonal)	++	-­
CD3 (Cell Marque; rabbit polyclonal)	-­	++
CD4 (4B12; bioGENEX; mouse monoclonal)	-­	+
CD5 (SP19; Cell Marque; rabbit monoclonal)	-­	++
CD8 (C8/144B; Cell Marque; mouse monoclonal)	-­	++
CD43 (MT1 ; Cell Marque; mouse monoclonal)	-­	+
CD99 (EPR3097Y; Cell Marque; monoclonal de conejo)	-­	++
TdT (Cell Marque; rabbit polyclonal)	-­	++
EBV (CS1-4; Cell Marque; mouse monoclonal)	-­	-
p53 (DO7; Cell Marque; mouse monoclonal)	-­	-
